# Transdisciplinary approaches to addressing factors that influence antimicrobial use in dairy cattle: A scoping review

**DOI:** 10.1016/j.heliyon.2024.e25550

**Published:** 2024-02-08

**Authors:** Jennifer Cole, Amtul Noor Mughal, Mahmoud Eltholth, Abin Thomas, Mark Holmes

**Affiliations:** a Department of Health Studies, School of Life Sciences and the Environment, Royal Holloway University of London, Egham Hill, Egham, Surrey, TW20 0EX, United Kingdom; b Global Academy of Agriculture and Food Security, The Royal (Dick) School of Veterinary Studies and the Roslin Institute, The University of Edinburgh, Edinburgh, United Kingdom; c Department of Veterinary Medicine, University of Cambridge, Cambridge, United Kingdom

## Abstract

Interest in antimicrobial resistance (AMR) associated with livestock farming is increasing. During the 1990s, 30–40 academic papers a year on the use of antibiotics in dairy farming were indexed on the scientific database PubMed, but this has grown to more than 200 a year in the 2020s. Most (85%) of these papers are published in veterinary or livestock science journals. There has been a corresponding increase in social science interest in why responsible antibiotic stewardship in the livestock sector is so challenging. However, most social science insights are published in journals specific to the lead authors’ field(s), missing opportunities for knowledge translation to veterinary and animal science. This threatens to inhibit the transdisciplinary One Health approaches required to tackle the problem. Between 1 June and 31 December 2021, we undertook a scoping review of papers on the use of antibiotics in dairy farming indexed in PubMed, Scopus and Web of Science. Our aim was to identify studies that incorporate social science approaches and methodologies, and to note the main field of the journal in which these studies are published. Papers were most likely to be published in veterinary science, dairy science and/or livestock science journals (61, 29 and 18 respectively out of 127 papers) and were most likely to be concerned with antibiotic use, prescribing practice, and/or diagnosis (94%, 39% and 33% of included papers respectively). Only 27% of papers meeting our inclusion criteria included a qualitative approach to understanding reasons for antibiotic use. Even fewer acknowledged underlying drivers of behaviour, whereas such reasons are frequently highlighted in social science literature. Thus, to address the global health threat from antibiotic resistance, more work is needed to bring together the disparate but equally valid disciplines, methodologies and researchers working on antibiotic use in the livestock sector.

## Highlights

• The number of papers on antibiotic use in livestock increased from 30 to 40 per year in the 1990s, to >200 per year today.
• Most papers are still predominantly focused on the Global North, but interest in LMICs is growing.
• Veterinary and animal science papers too often focus on which antibiotics are being used without fully considering why.
• Knowledge from social science in particular fails to reach all disciplines and fields, inhibiting a fully integrated approach.

## 1 Introduction

The welfare of animals, and the interconnection of human and animal health with one another and with the environment, attracts interest far beyond the field of veterinary science. The Manhattan Principles on “One World, One Health” [1] were drafted shortly after the turn of the 21st century and refreshed in the Berlin Principles on One Health [2] nearly two decades later. There is a growing awareness that the health of animals, humans and the environment is entangled [3,4].

Academics from diverse fields including geography (e.g. Refs. [[5], [6], [7]]), anthropology (e.g. Refs. [[8], [9], [10]]), economics (e.g. Refs. [11,12]), ethics (e.g. Refs. [13,14]) and religious studies (e.g. Ref. [15]) have joined veterinarians and animal scientists in seeking to understand the complex systems inhabited by Earth's living creatures. These systems are ‘socioecological’ (SES), a term coined by the Nobel prize-winning economist Elinor Ostrom [16], p1] to describe the linkages between social, economic and political settings and related ecosystems. This understanding is needed to explain, for example, the factors influencing why some agricultural systems are managed effectively but others less so, or why some farm environments are more polluted than others. Ostrom also warned, however, that while scientific knowledge is needed to, “enhance efforts to sustain SESs [ …] the ecological and social sciences have developed independently and do not combine easily.”

Recently, several academic programmes and networks dedicated to exploring human-animal-environment systems from socioeconomic *and* socioecological perspectives have arisen, including London School of Hygiene and Tropical Medicine's Anti-Microbials in Society (AMIS) programme [17], UK Economic and Social Research Council (ESRC)'s appointment of an Antibiotic Resistance champion for the social sciences [18], and the Microbes and Social Equity programme run by Sue Ishaq's lab at Maine University, USA [19]. Such initiatives bring social science – and social scientists – firmly into the One Health field but in most cases their work is published in journals specific to their own disciplines, not those more likely to be read by veterinarians who have much to learn from such transdisciplinary approaches [20]. This disconnect, observed across our involvement in two One Health projects (NEOSTAR^1^ and DARPI^2^) funded under a UKRI ESRC call to bring social sciences into AMR research^3^ [[21], [22], [23]], provided us with the incentive to explore the extent to which systematic and transdisciplinary approaches to understanding the reasons *why* antibiotics are used, rather than just quantifying their use, have been a feature of the literature on antibiotic use in dairy farming.

Ensuring that transdisciplinary findings are translated across disciplinary boundaries has been identified as critical not only by Ostrom but also by the fields of planetary health [[24], [25], [26], [27]] and EcoHealth [[28], [29], [30]]. One Health readily acknowledges the need to better integrate transdisciplinary research [4], stating: “in order to gain a deeper systemic knowledge of these interactions, we need sustainable collaborations and partnerships between human and veterinary medicine, social sciences and humanities, plant and environmental sciences, ecology, economics and many other relevant disciplines”.

The objectives of our review are firstly to quantify the extent to which social science is currently included in studies published in veterinary science and animal science journals; and secondly to highlight the themes that most often arise when social science is included. By highlighting the contribution that social science approaches and methodologies can bring, we hope to encourage veterinary and animal science researchers to work more closely with social scientists, and journal editors to seek out contributions from a more diverse range of authors.

## 2 Materials and methods

### 2.1 Search strategy

Between 1 June and 31 December 2021 we undertook a scoping review to determine the extent to which existing literature on the use of antibiotics in dairy farming includes social science. We began with two specific research questions: What are the **socioeconomic factors influencing antimicrobial use** in dairy cattle? and, What are the **management factors influencing antimicrobial use** in dairy cattle?

We added a third research question, which emerged frominformed our own work in India, carried out between 2018 and 2019: What are the **climatic/environmental factors influencing antimicrobial use** in dairy cattle? Discussions with farmers in Bangalore and Guwahati that sought to answer the first two questions had highlighted severe pressures on their livelihoods from climate change; this would have been missed had the projects not included social science methodology [31,32]. The discussions added an additional dimension to the explorations of underlying drivers of antimicrobial use.

To attempt to answer these three questions, we searched within three prestigious scientific and medical databases/indexes: PubMed, Scopus and Web of Science. Limited time and resources did not permit us to search more extensively but these three databases provide broad coverage of the available literature. We acknowledge the wide body of social science literature within social science journals, including by anthropologists (e.g Refs. [33,34]), human geographers (e.g. Ref. [35]), economists (e.g. Ref. [12]) and ethicists (e.g. Refs. [36,37]) but our aim was to identify the visibility of these approaches within veterinary and animal science literature specifically and the extent to which they appear in journals primarily produced for veterinarians and livestock scientists, not to conduct a systematic review across all the available literature from every field.

The search strategy follows the general guidelines for conducting a review published by the International Cochrane Collaboration (http://www.cochrane.org/index.htm), Centre for Reviews and Dissemination [38] and Sargeant and O'Connor [39]. We acknowledge that this is not a fully systematic review and does not follow the full protocols required of one. We made a conscious choice not to use the AMSTAR2 systematic review tool [40] for example, due to its primary focus on quantitative research and randomised controlled trials. Many of the papers we sought to assess do not match these criteria: indeed, we were actively looking for papers that are unlikely to. We also acknowledge that significant additional literature that meets our criteria has been produced since the conclusion of our review period, e.g. ([[41], [42], [43]]). We do not have the resources to fully incorporate these into later iterations of our original draft, but very much hope other researchers will do so in future.

We sourced articles exploring the factors influencing AMU in dairy farming outlined in our questions: socioeconomic, management, climate/environmental factors. The three selected electronic databases/indexes were searched for papers published up to and until 31 December 2021. We deliberately did not use search terms such as ‘social science’, ‘qualitative’ etc that would have immediately identified such papers, as we wanted to see how many emerged naturally from primarily veterinary, animal health and One Health literature. This would reveal the extent to which such methodologies and approaches are seen as an integral part of such literature. With limited time and resources available, we could not search every available database: the three we selected are well-respected, representative of and comprehensive for the veterinary, animal health and One Health sectors. We acknowledge that this is a limitation of the study. Additional literature is likely to be available that was missed by our search, in particular literature published in a language other than English and in country-specific journals that are not always deposited in international libraries.

The population of interest is dairy cattle, irrespective of breed, type of housing, geographic location, or calving distribution. The following combinations of search terms were applied: “dairy AND (cow* OR cattle OR bovine) AND (antibiotic OR antibiotics OR antimicrobial OR antimicrobials).” Search terms were broad to increase the sensitivity of the search result. References were uploaded to a web-based review management software programme (Covidence^4^).

### 2.2 Screening

**Fig. 1:**
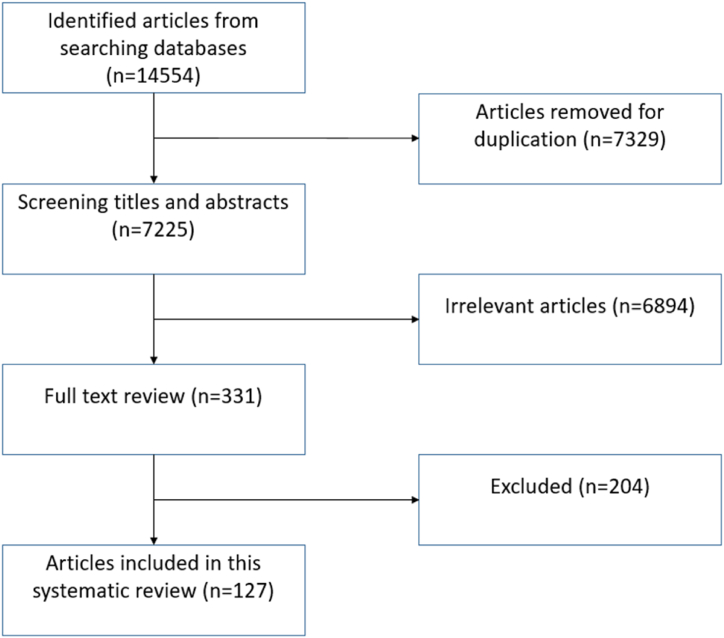
PRISMA diagram showing the identification and selection process of relevant articles.

Initially, 14,554 articles were retrieved from the database searches (See Fig. 1). After automatic removal of duplicates (n = 7329) by the Covidence software the titles and abstracts of 7225 articles were screened by two independent reviewers. For an article to pass this stage, it must have been published in the English language, and address at least one of the research questions above. Studies conducted on dairy cattle, farmers, veterinarians, or any relevant stakeholders associated with AMU or animal health were included for critical appraisal. A subjective judgement on what constituted a primarily relevant paper was made by the two independent reviewers and we accept this could be open to bias. Where there was disagreement on whether an article should be included, a third reviewer acted as arbiter. The full texts of articles considered to be primarily relevant by two reviewers (n = 331) were uploaded to the system. Primary papers reporting original data were included. The following articles were excluded: those not in English, reviews, letters, editorial articles, book chapters, conference papers and theses. After the selection process, only published data were used; authors were not contacted to provide any additional information. This process resulted in the inclusion of 127 articles for data extraction. A full list of the papers selected is provided [see: Supplementary material A]. Once included, papers were ordered by publication date in the database and assigned a Roman numeral identifier preceded by ‘P’ for ‘paper’ (e.g. Pvii is paper number 7). Throughout the rest of this paper, the Roman numeral identifier is used when referring to papers from the review to differentiate them from standard citations used as reference throughout the text.

### 2.3 Data extraction

To capture the required data from selected articles, the research team developed a data extraction form in Covidence. Data extraction was piloted by all investigators on five studies. Extracted data comprised: year of publication; country and/or region of study; field of journal in which the paper was published (determined by the journal's description of itself from information pages on its website); funding source(s); study methodology (quantitative, qualitative or semi-qualitative); the authors' main aims; conclusions/key findings and any recommendations for future research. The final three items were copied verbatim from the published text wherever possible. Data were extracted by one reviewer and independently checked by a second reviewer. Conflicts were resolved by a third reviewer.

### 2.4 Thematic analysis and data synthesis

The key findings of included articles were assessed for descriptive themes that aligned to the research questions we were seeking to answer: What are the **socioeconomic factors influencing antibiotic use** in dairy cattle? What are the **management factors influencing antibiotic use** in dairy cattle? And, What are the **climatic/environmental factors influencing antibiotic use** in dairy cattle? These were identified first by subjective analysis, by reading through the data extracted. Common themes that emerged were manually coded. Each paper was subjectively assessed either as ‘practice’ or ‘knowledge/attitudes’, depending on whether it focussed only on veterinary or farmer *practice* (how they used antibiotics, including the quantities and classes of antibiotics used) or also on *knowledge and attitudes* (how much veterinarians and farmers know about antibiotics and antibiotic resistance, and what drives or underlies their use of the drugs). The extracted text was copied to an ExCel database and this was then searched using keywords ‘manag’ (to allow for manage, management, managing) to identify those with a strong focus on management practices; ‘socio’ and ‘cost’ to identify those which had a strong focus on socioeconomic factors that might influence antibiotic use; climat (to allow for climate and climatic) and environ (to allow for environment and environmental) to identify those with a strong focus on environmental conditions. We acknowledge that searching within the full text of the articles may have identified some additional papers that met the inclusion criteria.

Next, the extracted text from the included papers was searched to quantify the number of times common themes observed during the subjective review – for example, ‘mastitis’ or ‘biosecurity’ – occurred in those papers. This process was carried out for all 127 included papers. The results are presented and analysed below. An ExCel sheet containing the extracted data that was searched for these terms is provided [see: Supplementary material A].

## 3 Results

A total of 127 papers met our inclusion criteria, the earliest of which was published in 1989. The number of papers on dairy cattle and antibiotics has increased year-on-year since then. Of the 127 papers included in our study, only two were published before 2000 (one in 1989 and one in 1996); 15 were published between 2000 and 2009; 64 were published between 2010 and 2019; and a further 25 were published in 2020 alone (See Supplementary Materials A and B). Just 17 of the 127 included papers (13%) were published before 2010.

**Fig. 2:**
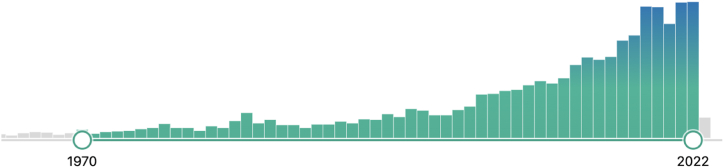
Papers returned by a search of Dairy Cattle + antibiotics on PubMed, up to 31 December 2021. © Pub med (https://pubmed.ncbi.nlm.nih.gov/?term=Dairy+cattle%2C+antibiotics%2C).

This is mirrored across the literature as a whole but as there is insufficient space in this paper to give a detailed breakdown of the growth of papers on all three databases individually, we use PubMed as an example. It is one of the world's most extensive scientific databases, indexing more than 30 million abstracts and citations dating back to 1809, with an additional 1 million + added each year. It thus provides a good overview of the publication landscape across all sectors and disciplines. The number of published papers indexed on PubMed has increased from around 30–40 a year in the latter part of the 20th century to more than 200 a year in the 2020s (see Fig. 2).

**Fig. 3:**
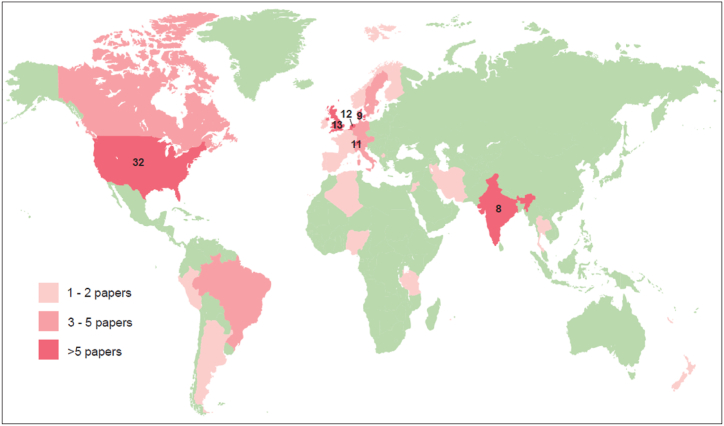
Map of the world showing countries where studies were conducted, and the numbers of studies for each country. The exact number is given for countries with >5 papers.

The 127 included papers represented studies undertaken in 28 countries across the world, covering the Global North and Global South, however the papers were not evenly distributed geographically as 74% were from the Global North and only 26% from the Global South. The percentage of papers relating to Europe, North America, South America, Asia, Middle East, Africa and Oceania was 45%, 28%, 8%, 7%, 2%, 1% and >1% respectively, with 5% having a global or unspecified coverage (see Fig. 3 and Supplementary Materials A and B).

These papers were published in journals from a range of fields (see Table 1). The primary field with which we classified each journal as being aligned was determined from that journal's description of its aims and purpose described on its own website. The represented fields were dominated by Veterinary Science (48%), Dairy/Livestock Science (23%), and Agricultural Science (14%) (see Table 2).

**Table 1:** Main field(s) of journals in which papers included in this study have been published.

Field	Number of papers
Veterinary science	61
Dairy science/Livestock science	29
Agriculture and biological sciences	18
Immunology and microbiology	9
Rural studies	2
Environmental science	2
Other	6 (1 paper each Cattle practice; Clinical practice; Zoonoses and public health; Dairy association; Infection, ecology and epidemiology; Medical sciences)
**Total**	127

**Table 2:** Themes and sub-themes covered by the papers, and research questions addressed.

Common themes and sub-themes covered by the papers			
Theme	Sub-theme	Number of papers	% of papers
**Veterinary action**	Treatment	120	94%
	Prescribing	50	39%
	Diagnosis	42	33%
**Diseases/conditions**	Mastitis	79	62%
	Dry cow	32	25%
	Respiratory	27	21%
	Foot conditions/Foot and Mouth Disease	16	13%
	Uterine conditions	12	9%
	Worms/parasites	9	7%
**Biosecurity/hygiene**	Cleaning practice	20	16%
	Biosecurity	14	11%
	Hygiene	13	10%
**Management**	At herd/farm level	82	65%
**Education**		37	29%
**Search terms used and number of papers returned for each term**			
**Research question**	**Search term**	**No of papers**	**% of papers**
**1**	Manag	98	77%
**2**	Cost	35	27%
**3**	Environ	26	20%
**2**	Socio	10	8%
**3**	Climat	3	2%

Of the 127 included papers, 92 (72%) were mainly concerned with recording use and practice of antibiotics only (*what* is being used), and 35 (28%) included at least some consideration of knowledge and/or attitudes towards that practice (*why* are antibiotics being used/used in this way?). Of the methodologies used across the papers, 83 (65%) were predominantly quantitative, 29 (23%) used predominantly qualitative approaches and a further 15 (12%) included some measure of qualitative approach to complement quantitative methods or vice versa, which we classified as ‘semi-qualitative’ (though few of these would be considered robust mixed methodology, particularly by social scientists). [See Supplementary Material A].

The term ‘treat’ or ‘treatment’ appeared in the extracted text of 120 of the papers (94%), ‘prescribe/prescription’ in 50 (39%) and ‘diagnose/diagnostics’ in 42 (33%). Mastitis – which is estimated to be responsible for 70% of all losses incurred by dairy farmers [44,45] – was mentioned in extracted text from 79 (62%) of all papers. Extracted text from 32 papers (25%) contained discussion of dry cow management or treatment. Respiratory conditions were mentioned in 27 (21%) of the papers' extracted text. Foot problems or foot and mouth disease (FMD) were mentioned in 16 papers (13%), uterine conditions in 12 (9%) and worms or parasites in nine papers' extracted text (7%). Nearly two-thirds of papers' extracted text (82 papers, 65%) mentioned the use of antibiotics for treatment and management at the herd/farm level.

### 3.1 Socioeconomic factors influencing antibiotic use in dairy cattle

With specific regard to the first research question, ‘What are the **socioeconomic factors influencing antibiotic use** in dairy cattle?‘, we searched within the extracted text of the selected papers using the terms ‘socio’, and ‘cost’ to identify those which had a strong focus on socioeconomic factors influencing antibiotic use. The term ‘socio’, to identify socioeconomic factors, appeared in 10 papers (Pxxiv, Pxxxi, Pxlviii, Plx, Plxiv, Plxviii, Plxxix, Plxxxi, Pxcvii, Pcv), of which six were from Europe, one from Africa (Nigeria), one from the USA and one from India. One additional paper modelled economic costs but not for not a specific region (Pcv). Seven of the papers were quantitative-only studies, and only three (Pxxiv, Plxviii and Pxcvii) had qualitative components. One (Pxxxi) was published in a medical journal that focussed more on human than animal health.

Thirty-five papers contained discussion of ‘cost’ as a factor in antibiotic use (Pii, Pxi, Pxvi, Pxxvi, Pxxix, Pxxxi, Pxxxii, Pxxxiv, Pxlv, Pliii, Plvi, Plix, Plxi, Plxvi, Plxv, Plxvi, Plxviii, Plxxv, Plxxviii, Plxxxi, Pxc, Pxciv, Pxcv, Pxcvii, Pxcviii, Pxcix, Pc, Pci, Pcxiii, Pcxvi, Pcxvii, Pcxix, Pcxxi, Pcxxiii, and Pcxxvi). Of these, 14 (40%) were qualitative or semi-qualitative studies.

The papers recognise the relationship between farm costs and antibiotic use and suggest that economic analysis or evidence-supported alternative approaches to animal husbandry are needed to support the introduction of better practices and to enable knowledge to be translated into practice. Regulation needs to present visible and tangible benefits to farmers in reducing their antibiotic use to ensure compliance. Farmers, particularly smallholders, operate within very tight profit margins and, when this is considered, their behaviour is often driven by rational action within the constraints they face (Pcxxvi).

Indeed, when the papers included in this study look further than the use of antibiotics alone, to the wider systems into which that use sits, cost is the most common structural barrier they find to improving use. Pcv highlights than when vets are cheaper than a dead cow, farmers will be more willing to use them, and Pxxxi makes the point that while the cost to human health associated with the sales of unpasteurised milk remains an externality to the farming industry, it might be better addressed through a robust cost benefit analysis that could help to drive government investment in veterinary services. Farmers may be aware that regulations require them to discard milk contaminated by bacteria or antibiotic residues, but often cannot afford to follow this course of action (e.g. Plxxxviii; Pcxxvi). Farmers often delay veterinary treatment due to cost [46]. Vets do not set out with the intention of misusing antibiotics but do so because of the immediate pressures on their livelihoods [23]. Farmers prefer to consult vets if they are able to do so: a veterinarian was the preferred choice of up to 80% of respondents (Pxcvii; Plxxxviii) and farmers who do consult them regularly are more likely to use antibiotics prudently – but professional veterinary services are often unaffordable (Pxxvi). Farmers often do not take their animals to a vet until they have tried other treatments first (Plxviii, Pcii); or rely on less qualified paravets because this is what they can more easily afford – this is particularly true of smallholder farmers (Plxxxviii). Pcii made the point that farmers would not consult a “quack” if a vet was available.

Pcix pointed to costs being a factor in which drugs vets choose to prescribe and Pcx quantifies this to 59% of vets saying price influences their decision. Cost also influences how farmers use the drugs they have been prescribed: Plxxxviii suggested that while farmers purchase the full course of drugs prescribed and report on quantitative surveys that they use the full course, observational social science methods reveal they often keep some back for future use.

### 3.2 Management factors influencing antibiotic use in dairy cattle

To answer the second research question, ‘What are the **management factors influencing antimicrobial use** in dairy cattle? we searched on ‘manag’ (to allow for manage, management, managing), which returned 98 papers – 77% of all papers included in the study. This is too many to list each one here individually, as we have for the other search terms. Discussion of disease management was common and was approached from several angles. For example, Pii discussed management of animal wellbeing and biosecurity as necessary to bring down disease risk and thus reduce antibiotic use. Pxvi linked disease management to herd size, recognising that risk is harder to manage as herd size increases. Pcx highlighted the differences between management practices on different sized farms, and that different approaches will be needed in different farming environments; this is consistent with social science literature from outside this study (e.g. Ref. [6]).

Biosecurity and shed hygiene are clearly seen as major components of livestock management and key ways to bring down antibiotic use: 20 papers' extracted text (16%) mentioned or criticised cleaning practices, 14 papers’ extracted text (11%) mentioned biosecurity and 13 (10%) mentioned hygiene.

Veterinarians routinely point to poor biosecurity and poor farm management as the main risk factors for disease (e.g. Plxviii and Plxxxviii). Such studies also acknowledge that biosecurity as it is understood in the Global North is not always practical in developing rural contexts. For example, Plxxxviii discusses how recommendations to isolate sick animals, house them separately and disinfect their shed, may not be possible for farmers whose animals live within their homestead and where space to do so is lacking; the study found that only 17% of farmers isolated sick animals and only 22% disinfected their sheds when illness was identified. The papers also reported that on smaller farms, the relationship between farmers and their animals is more personal and caring, whereas on larger farms it is more transactional; animal welfare tends to be higher on the smaller farms, at least until stricter regulation on animal welfare and living conditions on larger farms kicks in. The move to more intensive farming practices, which brings with it a drop in animal welfare, a corresponding jump in illness and therefore a greater requirement for antibiotics, is as a major issue considering the planned expansion of the dairy sector in developing regions.

The recognition of antibiotics as a “quick fix” for poor hygiene and other structural issues in systems that find the latter hard(er) to implement has been acknowledged in medical literature for the human health field [47,48] but on the results of our search is not yet as well represented within veterinary and animal management journals.

A move away from an assumption that bigger is better, towards better management of smaller-scale, more organic farms could be beneficial for keeping antibiotic use low. Such an approach could enable developing regions to ‘leapfrog’ the problematic highly intensive but with low welfare phase and take knowledge instead from small European and North American organic farming operations. Acknowledgement is also needed that animal health is not always improved on organic farms [49] and can be particularly problematic where hygiene is poor and over-crowding is high [50]. Papers included in the study on the post-intensive transition back to smaller, more ‘organic’ and more traditional methods (e.g. Pxix from Thailand) show that a shift away from intensive farming to more organic farms, particularly when combined with better access to veterinary services, does bring down antibiotic use, largely due to better herd health in general. Some papers (e.g Pcx) also suggest that proportionately far more antibiotics are used in large and medium-sized farms than on small ones, and milk from larger farms contains more antibiotic residues. While farmer knowledge and awareness of AMR and antibiotic stewardship also goes up with farm size, structural barriers on those farms can prevent the knowledge from being put into practice.

### 3.3 Climatic/environmental factors influencing antimicrobial use in dairy cattle

To answer the third research question, What are the **climatic/environmental factors influencing antimicrobial use** in dairy cattle? we searched on climat (to allow for climate and climatic) and environ (to allow for environment and environmental) to identify those with a strong focus on environmental conditions. Climate was mentioned in only three papers (Pviii, Plix and Plxix; 2%); Plxix discussed the effect of high temperatures and humidity on increased veal calf mortality; Pviii discussed shed management to deal with harsh winters in Wisconsin USA; and paper Plix considered the impact of climate on respiratory disease amongst cattle in Portugal. Awareness and evidence gathering on the systemic pressures from climate factors on disease risk and antibiotic use seems to be sorely lacking in the current literature.

‘Enviro[nment]’ was mentioned in 26 papers, but this was not usually in relation to the climatic environmental conditions but rather the farm environment such as sheds (e.g. Pxlii, Pxlvi, Pcx, Pcxxiii), environmental policy or management (e.g. Plxxix, Pcxv) or to the presence of antibiotic resistant bacteria in soil, water and wildlife (e.g. Pix). One paper (Pcxx) referred to “the influence of the social environment (i.e., positive references, awareness of what others say or do), the strength of marketing strategies of the pharmaceutical companies, previous experience” on antibiotic use, and (Pxcvii) also acknowledged that the relationships between stakeholders represents an important social environment that affects decision making. Regarding environmental change driven by climate trends, however, only three papers – Pviii, Plix and Plxix – genuinely considered the impact of weather patterns on disease risk and antibiotic use. Considering that many dairy farming regions of the world will come under increasing climate stress in coming decades [32], this is an area where more research is needed.

Two additional themes emerged from the analysis of data that are worth some consideration here: education, and the use of traditional treatments, such as indigenous medicinal herbs and homeopathic remedies from Ayurvedic and other non-allopathic Indian traditions [51].

### 3.4 Education

A significant number of papers (e.g. Pvi, Pxiii, Plxv, Pcxiv, Pcxvii) point to better farmer and/or veterinarian education on AMR and its dangers as a solution to suboptimal antibiotic use. Thirty-seven papers included in the study (29%) recommended more education, with roughly half suggesting vets, and half suggesting farmers (and often both) need more education. Vets, as well as researchers, bemoan farmers’ lack of education on AMU and AMR. This is despite farmers often having little agency in what drugs are available to them. Several papers recognise that most prefer to defer to a more knowledgeable veterinarian when possible (as our own fieldwork noted [23]), but there is often a shortage of veterinarians available to consult, and those that are available may not be affordable to smaller farmers. Furthermore, better education and tighter regulation does not always solve the problem. Antibiotic drug use remains both intensive and highly variable in Europe, where regulation is tighter (Plxxxv) and differs considerably between European countries subject to the same regulation (Plx), suggesting that there are considerable local and cultural factors at play in how antibiotics are used.

The calls for ‘more education’ that are still common in these scientific papers often fail to consider whether the infrastructure to improve the situation will be in place once farmers are ‘better educated’ on how to improve their practices. Pxxxi highlights the health risks from the sale of contaminated milk in Kosovan local markets but does not also consider how and where milk might be pasteurised, whether a road network to transport it from farm to pasteurisation plant and back to markets is in place, whether local markets and retailers have cold storage to keep pasteurised milk from spoiling, and whether that cold chain also extends back into consumers' homes; just telling people how dangerous it is to drink unpasteurised milk will not, alone, make the system safer.

Plxviii found that farmers often consider Community Extension programmes, aimed at embedding veterinarians in rural communities, to be more for the benefit of the veterinary students’ education than for the villages they are mean to serve. This suggests that more participatory action research with farmers and codesign of community programmes would be of benefit in future and should be a major part of antibiotic stewardship programmes going forward.

Education in better animal welfare and hygiene – as discussed above – on the other hand, may have more impact than education about antibiotics, as farmers have more agency to change this. Biosecurity and hygiene – of the farm environment, of the dairy sheds, of the milk the cows produce – and animal welfare (e.g. Pxxiv, Plx) often emerge as risk factors for antibiotic use, though insufficient attention is given to structural barriers that make the implementation of ‘better’ practices challenging even if the farmers receive education on how to improve this.

As Plxxvii acknowledges, a more effective approach than just telling farmers to do better would be to share positive outcomes of research trials to reduce antibiotic use, including economic advantages and improved profitability, improvements in herd health and evidence of low economic risks from improving animal welfare. Plxviii and Pxcvi both make clear that farmers will need clear cost benefit analysis of alternatives, such as better hygiene practices, spelled out to them if they are expected to adopt them on their farms.

### 3.5 Traditional treatments

Plxxxviii recorded that 90% of participants preferred allopathic treatments to more traditional ones and would pay for them if they were able to, but also raised the question of whether there needs to be more research on whether traditional treatments could have a larger role in addressing low level ill-health. Literature is beginning to emerge on the value of traditional medicine in global health systems [52], and there is some evidence that these treatments can be effective in bringing down antibiotic use [53,54]. The papers reported between 60% (Plxxxix) and 74% (Pcxxvi) of vets using ethnoveterinary practices such as Ayurveda and homeopathy alongside allopathic medicines, though 51% consider it less effective and only 20% think it could replace antibiotics completely (Plxxxix). Plxviii posited that ethnoveterinary practices are currently undervalued and that there is a lack of focus on these as a viable alternative to high antibiotic use and pharmaceutical interventions. Plxviii also questioned whether a tradition of treating one's own animals with herbal remedies instilled in farmers the idea that they could diagnose and treat themselves, and did not need vets, though this assumption was not tested in the study.

## 4 Discussion

Overall, the number of papers published on antibiotics in dairy farming has increased year-on-year in recent decades. Worldwide, there has been a five-fold increase in papers on antibiotics and dairy farming since the turn of the 21st century. This trend is mirrored in the papers included in our study and may be a reaction to the increasing international interest in AMR and imprudent antibiotic use as a global public health threat: 93 of the included papers (73%) have been published since the release of the O'Neill report in May 2016.

This increased interest in the importance of AMR as a global (human) health risk has brought with it a widespread but equally widely condemned (e.g. Ref. [55]) practice of blaming farmers and the livestock sector for overuse of antibiotics. This has driven significant research into quantifying the use of antibiotics in the sector and also a call for more systematic and transdisciplinary approaches to understand the reasons for that use (e.g. Refs. [56,57]). We found that only around a third of the papers that met our inclusion criteria contained any element of social science or qualitative evaluation (23% qualitative plus 17% semi-qualitative) however, suggesting that scientific papers are still not fully incorporating social science approaches. The vast majority of the research interest we did find was contained within veterinary science and agricultural practice journals: only one paper that met our inclusion criteria, (Pxxxi) was from a medical journal predominantly concerned with human health, one other (Pxiv) from a public health journal, and one (Pcii) from a general ecology and epidemiology journal. This suggests that understanding of how antibiotic use in livestock impacts on human health is not sufficiently crossing disciplinary and sector boundaries.

The importance of taking an holistic, systemwide approach to complex problems such as AMR is well-recognised within existing literature (e.g. Ref. [58]), as is the understanding that such an approach must consider humans and animals holistically (e.g. Refs. [4,59]; whilst our review was concluded before the publication of the latter paper, we must acknowledge both the paper itself and the recent attention given to One Health in *The Lancet* series of which it is a part).

Literature from across the world shows awareness that reducing antibiotic use will not be achieved by focussing on drug use alone (e.g. Plxxxv, which acknowledges that different farming systems will require different approaches). Some literature does acknowledge the wider socioeconomic system in driving animal ill-health, for example Plxix considers the impact of housing densities, shed climate and transportation of animals on disease risk, spread and the emergence of antibiotic resistance and Pcxl considers the inter-relatedness of livestock, waste management and the farm environment in enabling AMR to emerge and spread. Plx also identifies different practices in different systems – in this case at what stage veal calves are transported for fattening – as having significant impact on their health and thus the use of antibiotics. Such papers recognise what Hinchliffe and colleagues have described as “disease situations” [60] in which environmental conditions, such as poor hygiene, overcrowding and stress drive disease risk: addressing these, rather than simply focussing on the use of antibiotics will be needed to bring down antibiotic use. Pcxiv speaks of the importance of the “interconnections between people, animals, plants and their shared environments” and Pcx states: “The study aimed to go beyond just assessing farmer knowledge levels. By investigating the farmer, the farm environment and the product, milk, a broader, more cross-validated insight into antibiotic usage was hoped to be achieved”. In Pcxxvi, the veterinarians participating in the study emphasize that a data-driven interdisciplinary approach will be crucial for combating antimicrobial resistance. An acknowledgement of the need for a more systemwide approach is thus seeping into the global literature but such papers are the exception, not the rule.

## 5 Conclusions

Our review has shown a growing interest in antibiotic use, antibiotic resistance, and antibiotic stewardship in the veterinary and animal science literature, with papers on these topics increasing in frequency over the previous three decades. The literature shows a strong interest/focus on farm management practices, veterinary awareness, and farmer education (despite evidence of a lack of farmer agency to affect change), with nascent attention given to the role of climate change in stressing already fragile and precarious systems. Literature from North America and Western Europe predominates, with increasing literature devoted to India, in particular since the emergence of NDM-1. The literature does, however, tend to focus on isolated sections of the entire Socioecological System and value chain, preventing a full examination of the complex interdependencies that drive antibiotic mis(use) and give (in our opinion) insufficient consideration to the role of farmer agency, the importance of co-designing interventions with farmers, and of considering the relationship between climate stress and farmers’ economic stresses. While there is evidence of growing incorporation of other disciplines, including social sciences and earth science, into One Health research, such literature still tends to be siloed into discipline-specific journals, which prevents easy dissemination to and engagement with all of the disciplines that need to take an active role in understanding and influencing antibiotic rational use and stewardship. In this regard, we welcome recent advances such as the launching of the CABI One Health platform [61] and The Lancet series on Global Health Security [62,63], both of which have occurred since our review was carried out. There is nonetheless still a long way to go to ensure that disciplines beyond veterinary and animal science, and farmers themselves, are included in research teams. Their voices need to be heard in the evidence bases that inform antibiotic strategy and drive research agendas. This will help to ensure that not only the quantity and classes of antibiotics used are researched but the reasons for and drivers of that use are fully considered, and thus that interventions to change the current situation have the strongest chance of being implemented successfully.

## Ethics declarations

• This study was approved by the Human Ethical Review Committee, Royal (Dick) School of Veterinary Studies, University of Edinburgh, UK. Reference HERC_673_21, and Royal Holloway University of London Ethics Review Board, Reference ES/S000186/1
• All participants provided informed consent to participate in the study.

## Data availability

The data analysed in this study has been included as supplementary material. Any data not available through the supplementary materials will be made available on request from the corresponding author.

## CRediT authorship contribution statement

**Jennifer Cole:** Writing – review & editing, Writing – original draft, Supervision, Project administration, Methodology, Investigation, Funding acquisition, Data curation. **Amtul Noor Mughal:** Writing – review & editing, Writing – original draft, Investigation, Data curation. **Mahmoud Eltholth:** Writing – review & editing, Writing – original draft, Supervision, Project administration, Methodology, Formal analysis, Data curation, Conceptualization. **Abin Thomas:** Writing – review & editing, Investigation, Data curation. **Mark Holmes:** Supervision, Resources, Project administration, Funding acquisition.

## Declaration of competing interest

The authors declare that they have no known competing financial interests or personal relationships that could have appeared to influence the work reported in this paper.

## Footnotes

1 Does AMR in livestock contribute to AMR in people in NE India? An interdisciplinary study (https://gtr.ukri.org/projects?ref=ES%2FS000186%2F1).

2 Chicken or Egg: Drivers for Antimicrobial Resistance in Poultry in India (DARPI) (https://gtr.ukri.org/projects?ref=ES/S000216/1).

3 see: https://www.gov.uk/government/news/uk-india-working-together-to-address-antimicrobial-resistance.

4 Covidence. Covidence systematic review software. Melbourne, Australia. Available at www.covidence.org: Veritas Health Innovation, 2021.
