# Supplementary material for: Transdisciplinary approaches to addressing factors that influence antimicrobial use in dairy cattle: A scoping review
Source: Heliyon. 2024 Feb 8;10(4):e25550. doi: 10.1016/j.heliyon.2024.e25550 (PMC10877173; doi:10.1016/j.heliyon.2024.e25550)
Supplement: Multimedia component 2 [file mmc2.docx]

***Table 1: List of articles published in chronological order***

| **Paper ID** |  | **Year of Publication** | **City/State (if given)** | **Country (if given)** | **Continent** |
| --- | --- | --- | --- | --- | --- |
| **001** | i | **1986** | Ontario | Canada | North America |
| **002** | ii | **1996** | (South-eastern) | Brazil | South America |
| **003** | iii | **2000** | Michigan | USA | North America |
| **004** | iv | **2002** | - | Denmark | Europe |
| **005** | v | **2003** | - | Denmark | Europe |
| **006** | vi | **2003** | (Southern Highlands) | Tanzania | Africa |
| **007** | vii | **2004** | - | USA | North America |
| **008** | viii | **2005** | Wisconsin | USA | North America |
| **009** | ix | **2005** | Pennsylvania | USA | North America |
| **010** | x | **2006** | Wisconsin | USA | North America |
| **011** | xi | **2006** | Washington | USA | North America |
| **012** | xii | **2007** | - | Sweden | Europe |
| **013** | xiii | **2007** | - | France | Europe |
| **014** | xiv | **2007** | South Carolina | USA | North America |
| **015** | xv | **2008** | - | Finland | Europe |
| **016** | xvi | **2009** | - | USA | North America |
| **017** | xvii | **2009** | Ohio | USA | North America |
| **018** | xviii | **2010** | - | Denmark | Europe |
| **019** | xix | **2010** | - | Thailand | Southeast Asia |
| **020** | xx | **2011** | - | Denmark, Italy, Sweden, the UK, Netherlands and Switzerland. | Europe |
| **021** | xxi | **2012** | - | Denmark, Norway Netherlands, UK, Germany, Austria and Switzerland. | Europe |
| **022** | xxii | **2012** | Santa Fe | Argentina | South America |
| **023** | xxiii | **2012** | - | Denmark | Europe |
| **024** | xxiv | **2012** | - | Belgium | Europe |
| **025** | xxv | **2012** | - | USA | North America |
| **026** | xxvi | **2013** | - | France | Europe |
| **027** | xxvii | **2013** | - | Sweden | Europe |
| **028** | xxviii | **2014** | - | Peru | South America |
| **029** | xxix | **2014** |  | | Global |
| **030** | xxx | **2015** | (Northern) | Spain | Europe |
| **031** | xxxi | **2015** | - | Kosovo | Europe |
| **032** | xxxii | **2015** | - | Netherlands and Germany | Europe |
| **033** | xxxiii | **2015** | Ontario | Canada | North America |
| **034** | xxxiv | **2015** | - | England and Wales | Europe |
| **035** | xxxv | **2015** | - | Argentina | South America |
| **036** | xxxvi | **2016** | - | Switzerland | Europe |
| **037** | xxxvii | **2016** | - | Brazil | South America |
| **038** | xxxviii | **2016** | Umbria | Italy | Europe |
| **039** | xxxix | **2016** | - | The Netherlands | Europe |
| **040** | xl | **2016** | - | Dutch Region | Europe |
| **041** | xli | **2016** |  | USA | North America |
| **042** | xlii | **2016** | - | Dutch Region | Europe |
| **043** | xliii | **2016** | - | Sweden | Europe |
| **044** | xliv | **2016** | Flanders | Belgium | Europe |
| **045** | xlv | **2016** | - | Netherlands | Europe |
| **046** | xlvi | **2017** | - | UK and Netherlands | Europe |
| **047** | xlvii | **2017** | - | | Global |
| **048** | xlviii | **2017** | - | Switzerland | Europe |
| **049** | xlix | **2017** | - | Austria | Europe |
| **050** | l | **2017** | - | Dutch Region | Europe |
| **051** | li | **2017** | - | UK | Europe |
| **052** | lii | **2017** | - | USA | North America |
| **053** | liii | **2017** | - | | Global |
| **054** | liv | **2017** | - | England | Europe |
| **055** | lv | **2017** | - | England | Europe |
| **056** | lvi | **2017** | - | New Zealand | Oceania |
| **057** | lvii | **2017** | Ontario | USA | North America |
| **058** | lviii | **2018** | Governate of Biskra | Algeria | North Africa |
| **059** | Lix | **2018** | - | Denmark, Portugal, Switzerland | Europe |
| **060** | lx | **2018** | - | Denmark, Portugal, Switzerland | Europe |
| **061** | lxi | **2018** | California | USA | North America |
| **062** | lxii | **2018** | Tennessee | Tennessee, USA | North America |
| **063** | lxiii | **2018** | - | Dutch Region | Europe |
| **064** | lxiv | **2018** | - | | Global |
| **065** | lxv | **2018** | - | Jordan | Middle East |
| **066** | lxvi | **2018** | - | UK | Europe |
| **067** | lxvii | **2018** | - | Netherlands | Europe |
| **068** | lxviii | **2018** | - | India | South Asia |
| **069** | lxvix | **2019** | - | Switzerland | Europe |
| **070** | lxx | **2019** | - | | Global |
| **071** | lxxi | **2019** | Tennessee | USA | North America |
| **072** | lxxii | **2019** | - | UK | Europe |
| **073** | lxxiii | **2019** | - | Austria | Europe |
| **074** | lxxiv | **2019** | Saskatchewan | Canada | North America |
| **075** | lxxv | **2019** | - | Switzerland | Europe |
| **076** | lxxvi | **2019** | - | | Global |
| **077** | lxxvii | **2019** | New York | USA | North America |
| **078** | lxxviii | **2019** | - | UK | Europe |
| **079** | lxxix | **2019** | (North-Central) | Nigeria | Africa |
| **080** | lxxx | **2019** | - | USA | North America |
| **081** | lxxxi | **2019** | - | USA | North America |
| **082** | lxxxii | **2020** | - | Austria | Europe |
| **083** | lxxxiii | **2020** | - | USA | North America |
| **084** | lxxxiv | **2020** | - | Germany, Italy, United States | Europe and North America |
| **085** | lxxxv | **2020** | - | | Global |
| **086** | lxxxvi | **2020** | - | England | Europe |
| **087** | lxxxvii | **2020** | Tamil Nadu | India | South Asia |
| **088** | lxxxviii | **2020** | Punjab | India | South Asia |
| **089** | lxxxix | **2020** | (Southern) | India | South Asia |
| **090** | xc | **2020** | Fribourg | Switzerland | Europe |
| **091** | xci | **2020** | (Central) | Italy | Europe |
| **092** | xcii | **2020** | - | USA | North America |
| **093** | xciii | **2020** | - | USA | North America |
| **094** | xciv | **2020** | - | Switzerland | Europe |
| **095** | xcv | **2020** | - | Germany | Europe |
| **096** | xcvi | **2020** | New Quebec | Canada | North America |
| **097** | xcvii | **2020** | - | Switzerland | Europe |
| **098** | xcviii | **2020** | - | USA | North America |
| **099** | xcix | **2020** | - | Brazil | South America |
| **100** | c | **2020** | New York | USA | North America |
| **101** | ci | **2020** | - | Denmark | Europe |
| **102** | cii | **2020** | - | India | South Asia |
| **103** | ciii | **2021** | (Western) | USA | North America |
| **104** | civ | **2021** | - | Switzerland | Europe |
| **105** | cv | **2021** | - | UK | Europe |
| **106** | cvi | **2021** | - | Peru | South America |
| **107** | cvii | **2021** | California | USA | North America |
| **108** | cviii | **2021** | California | USA | South America |
| **109** | cvix | **2021** | Punjab | India | South Asia |
| **110** | cx | **2021** | Assam, Haryana | India | South Asia |
| **111** | cxi | **2021** | - | Switzerland | Europe |
| **112** | cxii | **2021** | - | Denmark | Europe |
| **113** | cxiii | **2021** | - | Canada | North America |
| **114** | cxiv | **2021** | - | Tanzania | Africa |
| **115** | cxv | **2021** | - | Iran | Middle East |
| **116** | cxvi | **2021** | California | USA | North America |
| **117** | cxvii | **2021** | (North-Westerrn) | Nigeria | Africa |
| **118** | cxviii | **2021** | - | USA | North America |
| **119** | cxix | **2021** | - | Netherlands | Europe |
| **120** | cxx | **2021** | - | UK | Europe |
| **121** | cxxi | **2021** | - | Ireland | Europe |
| **122** | cxxii | **2021** | - | Canada | North America |
| **123** | cxxiii | **2021** | New York | USA | North America |
| **124** | cxiv | **2021** | - | Italy | Europe |
| **125** | cxv | **2021** | New York | USA | North America |
| **126** | cxvi | **2021** | - | India | South Asia |
| **127** | cxvii | **2022** | Santa Catarina | Brazil | South America |
